# Excipient-Free Pure Drug Nanoparticles Fabricated by Microfluidic Hydrodynamic Focusing

**DOI:** 10.3390/pharmaceutics13040529

**Published:** 2021-04-10

**Authors:** Roni Sverdlov Arzi, Asaf Kay, Yulia Raychman, Alejandro Sosnik

**Affiliations:** 1 Laboratory of Pharmaceutical Nanomaterials Science, Department of Materials Science and Engineering, Technion-Israel Institute of Technology, 3200003 Haifa, Israel; ronisv@campus.technion.ac.il (R.S.A.); jul89ray@gmail.com (Y.R.); 2 Laboratory of Electrochemical Materials and Devices, Department of Materials Science and Engineering, Technion-Israel Institute of Technology, 3200003 Haifa, Israel; asaf.kay@gmail.com

**Keywords:** kinase inhibitors, pure drug nanoparticles, drug nanocrystals, bottom-up nanonization, nanoprecipitation, microfluidics, flow focusing technologies

## Abstract

Nanoprecipitation is one of the most versatile methods to produce pure drug nanoparticles (PDNPs) owing to the ability to optimize the properties of the product. Nevertheless, nanoprecipitation may result in broad particle size distribution, low physical stability, and batch-to-batch variability. Microfluidics has emerged as a powerful tool to produce PDNPs in a simple, reproducible, and cost-effective manner with excellent control over the nanoparticle size. In this work, we designed and fabricated T- and Y-shaped Si-made microfluidic devices and used them to produce PDNPs of three kinase inhibitors of different lipophilicity and water-solubility, namely imatinib, dasatinib and tofacitinib, without the use of colloidal stabilizers. PDNPs display hydrodynamic diameter in the 90–350 nm range as measured by dynamic light scattering and a rounded shape as visualized by high-resolution scanning electron microscopy. Powder X-ray diffraction and differential scanning calorimetry confirmed that this method results in highly amorphous nanoparticles. In addition, we show that the flow rate of solvent, the anti-solvent, and the channel geometry of the device play a key role governing the nanoparticle size.

## 1. Introduction

Nanotechnology has made a significant contribution to overcome (bio)pharmaceutical drawbacks of drugs such as poor aqueous solubility, low physicochemical stability in the biological milieu, short half-life and low bioavailability and efficacy [1,2,3,4]. For instance, >60% of the approved small-molecule drugs and ~90% of new drugs under development are classified as poorly water-soluble according to the Biopharmaceutics Classification System [5,6,7,8]. These drawbacks challenge the translation of drug candidates into new products, contribute to the high drug attrition rates in pharmaceutical development, and motivate the pharmaceutical industry to seek for non-traditional dosage forms and delivery routes [9,10,11,12,13,14].

Nanonization via top-down and bottom-up techniques has gained clinical impact [1,15,16,17,18,19,20] to increase the dissolution rate and saturation solubility of pure drugs due to reduction of particle size and the associated increase of the specific surface area-to-volume ratio [15,21,22,23]. Top-down techniques involve the breakdown of large particles into smaller ones by mechanical forces (e.g., high pressure homogenization, wet ball milling) [19,20], whereas bottom-up techniques (e.g., nanoprecipitation, sono-crystallization, and drying technologies) produce particles through precipitation from a solution at the nanometer scale. The former methods are straightforward and reliable for industrial scale-up. Conversely, the latter offer greater flexibility during the synthesis as well as improved control over the physicochemical characteristics of the product (e.g., particle size, morphology, amorphousness versus crystallinity) by adjusting the process conditions [10,16,24,25]. Additionally, bottom-up techniques enable the combination of more than one active pharmaceutical ingredient (API) in one single nanoparticle such as in the case of drug-drug co-crystals [26,27].

Amid the nanotechnology-based products that have already been approved by U.S. Food and Drug Administration (FDA) for clinical use, pure drug nanoparticles (PDNPs), within a size range from a few nanometers up to 1 μm, represent the simplest and one of the most patented technologies to increase the water dissolution rate of hydrophobic drugs and increase their oral bioavailability [28,29,30,31,32]. PDNPs have also been used in the development of long-acting injectable formulations [33] and their ability to increase the adhesion to the intestinal mucosa and prolong their residence time in the gut with respect to microparticles has been reported [16,29]. In addition, the use of PDNPs for targeted drug delivery by the intravenous route has been explored [34].

One of the most straightforward bottom-up techniques to produce these particles is by liquid anti-solvent (AS) precipitation that enables the fast, simple and energy-efficient formation of a wide range of nanomaterials [1,30,35,36,37,38]. However, the successful translation of nanoparticle formulations using conventional nanoprecipitation techniques still faces challenges, including the difficulty to control the size and the size distribution of the particles, their physicochemical instability in suspension (as they tend to agglomerate), and the batch-to-batch variability due to the lack of control over the mixing process in the solution bulk [37,39,40]. The incorporation of colloidal stabilizers (e.g., surfactants) is often required to control the drug particle growth and prevent particle agglomeration and thus, increase the physical stability of the nanosuspensions [1,41,42,43,44]. The incorporation of surfactants in the production process results in a smaller amount of API in the final product [34] and, in some cases, they have been associated with toxicity and side-effects [4,27].

Microfluidics emerged as a powerful tool in biology and nanomedicine, in general, [45,46,47,48] and pharmaceutical sciences, in particular, to synthesize surfactant-free PDNPs with controlled size and improved physical stability by the manipulation of fluids in micrometric channels/capillaries networks [39,40,49,50,51,52,53]. The precipitation process takes place inside the channels where the solvent (S) and AS are rapidly mixed, allowing precise liquid handling and uniform mass transfer, which in turn enables superior control over the features of the produced particles [22,24,37,54,55,56]. The mixing rate inside the channels is determined by the diffusion rate of the molecules across the interface and between the two fluids subjected to a continuous flow [45,51,57]. Another advantage of microfluidics is that the amount of reagents used for synthesis is very small, making this platform extremely cost-effective, especially in early pharmaceutical research and development stages [40,58]. Owing to the controlled nature of this technique, particle size can be optimized by changing the conditions of the precipitation process (e.g., channel geometry, precursor composition, flow rate) [27,59]. Several works in the field of nanomedicine demonstrated the advantage of using microfluidics for the production of drug-loaded polymeric and lipid microparticles and nanoparticles, although very scarce research has been dedicated to investigate this technology in the synthesis of excipient-free PDNPs [22,60,61].

Protein phosphorylation is the most common form of reversible post-translational modification and it is controlled by kinases [62]. Kinase signaling pathways have been shown to drive many of the hallmark phenotypes of tumor biology, including proliferation, survival, motility, metabolism, angiogenesis, and evasion of antitumor immune responses. Kinase inhibitors belong to the so-called molecularly-targeted anticancer therapies and they emerged as one of the most intensively pursued targets [63,64]. To date, >40 small-molecule kinase inhibitors have been approved by the FDA for the therapy of cancer and, more recently, of autoimmune diseases such as rheumatoid arthritis and inflammatory bowel diseases [65]. In addition, >100 compounds are under clinical trials [63]. Many kinase inhibitors display poor aqueous solubility and moderate to low oral bioavailability which jeopardizes their pharmacokinetics [66]. Others display a pH-dependent dissolution profile that results in differential precipitation along the gastrointestinal tract. Kinase inhibitors are administered by the oral route, although a few studies proposed their use also by injection for local therapy [67,68]. Nanonization of pure kinase inhibitor nanoparticles emerges as a clinically relevant technological strategy, not only to improve their oral bioavailability but to also take advantage of alternative administration routes (e.g., intranasal) to target brain tumors [69,70].

In this study, we report on the design and fabrication of simple, cheap, and mechanically stable Y- and T-shaped Si-made microfluidic devices by using photolithography and demonstrate their use to produce surfactant-free and physically stable, pure, and highly amorphous nanoparticles of three kinase inhibitors, namely imatinib (IMA), dasatinib (DAS) and tofacitinib (TOF), via hydrodynamic focusing.

## 2. Materials and Methods

### 2.1. Materials

IMA free base and DAS free base monohydrate were supplied by Carbosynth Ltd. (Compton, UK) and TOF free base by LC Laboratories (Woburn, MA, USA). Ethanol, isopropanol, and acetone were purchased from Bio-Lab Ltd. (Jerusalem, Israel). Milli-Q water was obtained from a Barnstead Smart2Pure 12L UV/UF water purification system (Thermo Electron LED GmbH, Niederelbert, Germany). All the solvents were of analytical and spectroscopic grade and were used as received.

### 2.2. Components of the Microfluidic System

Silicon wafer was purchased from UniversityWafer, Inc. (Boston, MA, USA), polytetrafluoroethylene tubing from Wirtham Marketing & Suppliers (Haifa, Israel), and AZ 4533^®^ and AZ 4562^®^ photoresists from MicroChemicals GmbH (Ulm, Germany). The mask made of quartz coated with a thin layer of chromium was designed by using AutoCAD^®^ 22.0 software (Autodesk, Inc., San Rafael, CA, USA).

### 2.3. Methods

#### 2.3.1. Design and Assembly of the Microfluidic Device

**Figure 1:**
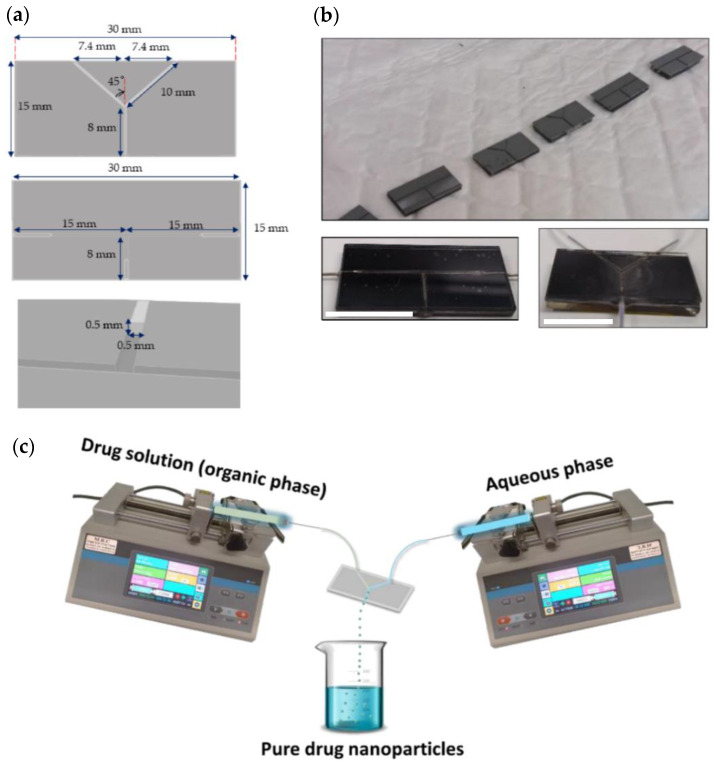
(**a**) Scheme of the T- and Y-shaped Si-made microfluidic devices and their dimensions; (**b**) The T- and Y-shaped microfluidic devices after fabrication (**upper**) and after the assembly of the three layers (**lower**). Scale bar = 15 mm; (**c**) Experimental setup using a Y-shaped microfluidic device. A similar process was conducted with a T-shaped chip.

The mask for the microfluidic channels was assembled using a computer-aided design and drafting software AutoCAD^®^. The width and depth of the channels were 500 µm, with two different channel configurations, T- and Y-shaped. The size of the microfluidic chips was 30 × 15 mm, the length of the inlet and outlet channels in the T-shaped device were 15 and 8 mm, respectively, while in the Y-shaped one, they were 10 and 8 mm (Figure 1a).

For the lithographic process, a piece of p-type Si wafer <100> was sliced, cleaned by sequential immersion in acetone, isopropanol, and water, and dried in dry air. Before dicing the wafer to its final dimensions (9.5 × 9.5 cm), the substrate was coated with AZ 4533^®^ photoresist to protect the mask. Then, the substrate was cleaned and spin-coated to apply a thin layer of AZ 4562^®^ photoresist by centrifugal force. After evaporation of the solvents in the photoresist via soft baking, the substrate was exposed to UV light through the mask with the desired microfluidic pattern. After baking, the exposed layer of the photoresist was removed by using chemical bath development. Finally, dry reactive ion etching was performed to generate the channels and the Si piece was diced into rectangles and further cleaned. The system was assembled into three layers: the upper and lower layers were made of glass for mechanical strength and transparency and the middle layer was made of a Si wafer with the embedded T- and Y- shaped channels (Figure 1b). Si was chosen owing to its good chemical compatibility and excellent thermal and mechanical stability under flow and pressure [71]. The top layer was glued using Epo-Tek^®^ 301 (Epoxy Technology Inc., Bill Rica, MA, USA) and the bottom and middle layers were glued together using Araldite^®^ (Basel, Switzerland).

#### 2.3.2. Production of Pure Additive-Free Kinase Inhibitor Nanoparticles

The overall experimental device consisted of a Si-made chip, two continuous infusion pumps (SYP-01, MRC Ltd., Holon, Israel) for supplying the drug solution in ethanol and water (used as AS), and a unit for the collection of the PDNPs in suspension. The setup is schematically illustrated in Figure 1c. To produce the PDNPs, each pristine drug was dissolved in ethanol (1 mL, final drug concentration was 0.1% *w/v*). Ethanol was chosen as the S because it dissolves well the three drugs, it is miscible with the AS and it can be eliminated by evaporation at room temperature (RT) and atmospheric pressure. Moreover, ethanol is classified as Class 3 solvent (solvents with low toxic potential) and is regarded as safe in relatively high amounts (daily exposure of up to ~50 mg/day) by the International Conference on Harmonization [72]. Next, the drug solution (called organic phase) and water (called aqueous phase) were injected by two syringe infusion pumps into the channels of a T- or Y-shaped microfluidic device and mixed rapidly in the intersection point at RT to produce the nanoparticles. The size of the particles was controlled by varying the following process conditions: channel geometry, the overall flow rate of each phase and the S/AS volume ratio. Once the precipitation process was completed, the nanosuspension was immediately frozen at −80 °C and freeze-dried (Labconco Free Zone 4.5 plus L Benchtop Freeze Dry System, Labconco, Kansas City, MO, USA) for further characterization.

#### 2.3.3. Characterization of Pristine Drugs and Pure Drug Nanoparticles

The hydrodynamic diameter (D*_h_*), the polydispersity index (PDI, an estimation of the particle size distribution) and the zeta-potential (Z-potential) were determined in a Zetasizer Nano-ZS (Malvern Instruments, Malvern, UK) at 25 °C with a 4 mW He–Ne laser (*λ* = 633 nm), a digital correlator ZEN3600 and Non-Invasive Back Scatter (NIBS^®^) technology at a scattering angle of 173° to the incident beam. D*_h_* and PDI were measured by using the dynamic light scattering (DLS) technique. DLS data were analyzed using CONTIN algorithms (Malvern Instruments). Z-potential analysis used of laser Doppler microelectrophoresis in the same instrument. Values are expressed as mean ± standard deviation (S.D.) and each measurement is a result of at least five runs. The S.D. of each size population, which is an expression of the peak width, was also determined. Differences among particle sizes were analyzed using one-way analysis of variance (ANOVA, significance level of 1%) with Bonferroni test (*p* < 0.01).

The morphology of the different PDNPs was visualized by high resolution-scanning electron microscopy (HR-SEM, carbon coating, acceleration voltage of 1–4 kV, Ultraplus, Zeiss, Oberkochen, Germany). For HR-SEM, the pristine drugs and PDNP suspensions were dispersed in water and sprayed on top of a p-doped Si wafer <100> by introducing high-pressure N_2_, allowing the individual particles to be spread evenly on the wafer. Then, the wafer was attached to the grid using carbon-tape and additional tape was placed on its frame. Silver paint (Structure Probe, Inc., West Chester, PA, USA) was applied to the corners of the frame prior to carbon coating.

The structure (crystalline versus amorphous) of the PDNPs was analyzed by powder X-ray diffraction (PXRD) in an XRD diffractometer MiniFlex (Rigaku, Tokyo, Japan) under parallel-beam geometry at a speed rate of 6° min^−1^, θ–2θ range of 5–50° (with intervals of 0.01°) on a poly(methyl methacrylate) slide, at RT. Diffractograms of the PDNPs were compared to those of the pristine drugs.

Thermal characterizations were performed by differential scanning calorimetry (DSC, 2 STARe system equipped with a simultaneous thermal analyzer, STARe Software V13 and intra-cooler Huber TC100, Metter Toledo, Schwerzenbach, Switzerland). For this, samples (5–10 mg) sealed in 40 µL-Al crucible pans (Mettler Toledo) were heated from 25 to 325 °C at a heating rate of 10 °C min^−1^ under N_2_ gas flow (20 mL min^−1^) and In was used as a standard.

## 3. Results and Discussion

### 3.1. Rationale

Our microfluidic devices were designed to promote passive mixing (i.e., mixing without the influence of external forces) which depends only on the flow rates and the geometry of the micron-sized channels. The flow regime and mass transfer mechanisms involved in the mixing of fluids in the channels are characterized by the dimensionless Reynolds number (Re) and Péclet number (Pe), respectively [73,74]. Re expresses the ratio between the fluid inertia and the viscous shear force and is defined by Equation (1).

(1) Re=ρVdμ

where ρ and μ are the density and the dynamic viscosity of the fluid, V is the speed that is representative of the flow and d is the channel characteristic length. Since the cross-section of our microchannels is rectangular, the characteristic length is calculated as the hydraulic diameter ($D_{H}$), defined by Equation (2).

(2) $$D_{H}=\frac{4A}{P}$$

where A is the cross-sectional area of the channel and P is the wetted perimeter. In this case, the wetted perimeter is the same as the cross-section perimeter, as the channel is filled by the fluid.

In general, small Re values (<2100) indicate laminar flow, while greater numbers indicate a turbulent flow regime [73].

Pe is defined as the ratio between the advective transport rate (Vd) of a physical quantity and the mass diffusion coefficient (D) of the same quantity driven by an appropriate gradient. In the context of mass transfer, Pe is the product of Re and Schmidt number (Sc), which is defined as the ratio between the kinematic viscosity (ν) and the mass diffusivity (D), as expressed by Equation (3).

(3) Pe=VdD=Re × Sc

Considering that in our systems, the diameter of the channel is in the order of micrometers (the height and width of the channels are 500 μm) and that water is the predominant phase (in terms of volume) contributing to the overall flow, the calculated Re values in our experiments are small enough (from 16.7 to 266.7 for a flow rate between 0.5 and 8 mL min^−1^, respectively, as calculated by using Equations (1) and (2)) to result in laminar flow of ordered streamlines. Thus, it is reasonable to assume that the main mass transfer mechanism inside the channels is provided by molecular diffusion. Laminar flow is desirable to allow improved control over the droplet size and consequently, the size of the PDNPs produced during the nanoprecipitation process [75].

**Figure 2:**
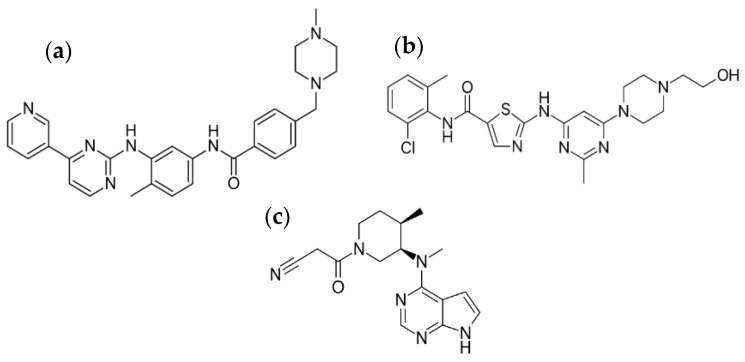
Chemical structure of (**a**) imatinib, (**b**) dasatinib and (**c**) tofacitinib (as free base).

To investigate the robustness of our microfluidic device for the synthesis of surfactant-free pure kinase inhibitor nanoparticles, we selected three compounds (in free base form) with a broad range of octanol–water partition coefficient (logP) values and intermediate to low intrinsic water solubility (S_0_) at neutral pH and 25 °C. IMA (S_0_ = 2 µg mL^−1^, calculated logP = 4.53, Figure 2a) [76,77] was the first FDA-approved kinase inhibitor that targets the Bcr-Abl tyrosine kinase and the phosphorylation of the platelet derived growth factor receptor and it is used for the treatment of chronic myeloid leukemia. Its oral bioavailability is 98% [64]. DAS (S_0_ = <1 µg mL^−1^, logP = 3.83, Figure 2b) [76,78] is a dual Bcr-Abl and Src tyrosine kinase inhibitor used in chronic myelogenous leukemia and acute lymphoblastic leukemia with an oral bioavailability of 14% and 34% in mouse and dog, respectively [79]. Intravitreal DAS injection has been proposed in the treatment of ocular diseases [67,80]. TOF (S_0_ = <300 µg mL^−1^, calculated logP = 1.19, Figure 2c) [76,78,81] is a Janus kinase inhibitor approved for the treatment of rheumatoid and psoriatic arthritis and ulcerative colitis and displays an oral bioavailability of 74% [82,83].

Pure kinase inhibitor nanoparticles were produced, and the effect of the physicochemical properties and the process conditions comparatively characterized. Initially, each pristine drug was dissolved in ethanol (S) and the drug solution and water (AS) were pumped via two infusion pumps into the two inlets of the microfluidic device and rapidly mixed inside the intersection point to ensure the controlled formation of the nanoparticles (Figure 1c). To reach a yield of ~100%, products were not filtered after the nanoprecipitation. Thus, the production of particles with size at the nanometer scale and small PDI was required. In addition, to maximize the drug content in the final product, we produced the nanoparticles without colloidal stabilizers that are used as pharmaceutical excipients to minimize the free energy of the colloidal system. This was challenging because PDNPs tend to aggregate in suspension and grow over time at relatively fast rates [9,84].

After the nanoprecipitation process, fresh samples were characterized by DLS. In addition, sample fractions were frozen at −80 °C and freeze-dried for characterization by PXRD and DSC.

### 3.2. Production and Characterization of Additive-Free Pure Kinase Inhibitor Nanoparticles

**Figure 3:**
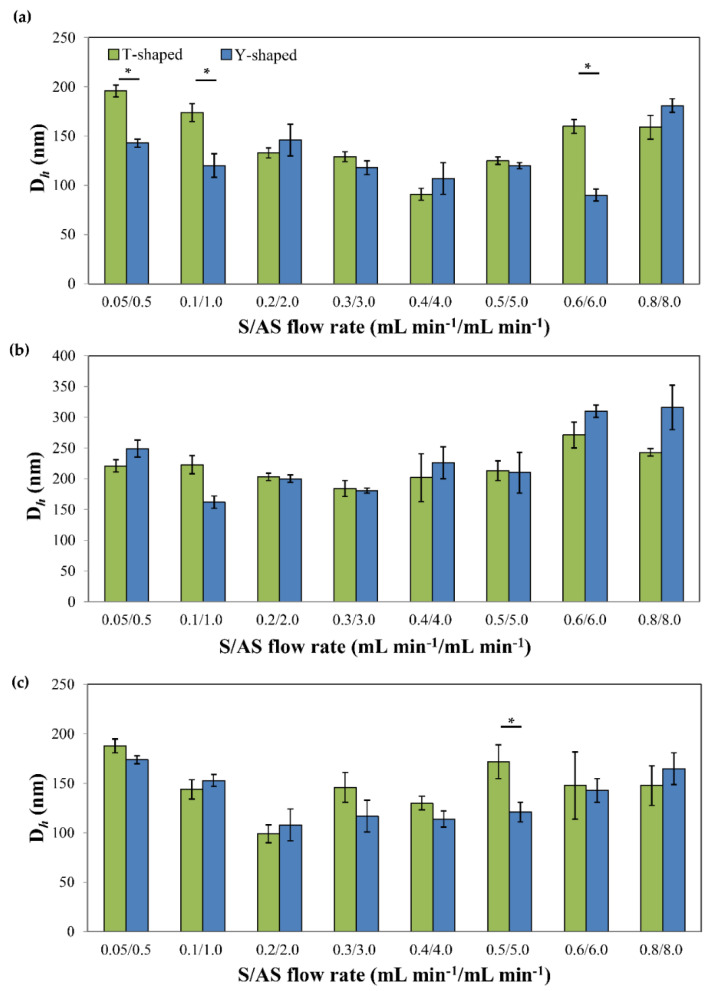
The effect of S/AS flow rate changes on the hydrodynamic diameter (D*_h_*) of excipient-free pure (**a**) imatinib, (**b**) dasatinib and (**c**) tofacitinib nanoparticles produced by using T- and Y-shaped devices, as measured by DLS at 25 °C. A constant S/AS volume ratio of 1/10 was used. * Denotes statistically significant difference in the D*_h_* between the two channel geometries (*p* < 0.01).

Initial studies focused on the effect of different system parameters on particle size and size distribution using the Y- and T-shaped microfluidic devices to optimize it. In this framework, the effect of the S and AS flow rates and the variation in the AS flow rate at constant S flow rate on the particle size were assessed. Results are summarized in Table 1 and Figure 3. The drug concentration of all drugs in ethanol was 0.1% *w/v* and we kept constant the S/AS volume ratio (1/10). All the experiments were conducted at 25 °C.

Following the change in the overall flow rate of the S and AS, the D*_h_* of the PDNPs was in the 80–200 nm, 170–350 nm and 90–190 nm range for IMA, DAS and TOF, respectively (Table 1). DLS results showed that at the limits of low and high S/AS flow rates, the D*_h_* of the particles was larger than that obtained at intermediate flow rates. For example, the D*_h_* of pure IMA nanoparticles produced in the T- and Y-shaped devices decreased from 196 ± 6 and 143 ± 4 nm to 91 ± 6 and 107 ± 16 nm at flow rates of 0.05/0.5 and 0.4/4.0 mL min^−1^/mL min^−1^, respectively (Figure 3, Table 1). A similar trend was observed for DAS and TOF nanoparticles. This trend could be explained by the delicate interplay between efficient versus incomplete mixing of the S and the AS during the precipitation process. Micro-mixing (i.e., mixing at the molecular scale) is a key factor in determining the degree of the supersaturation of the drug and its local spatial distribution [57]. Subsequently, when the overall flow rate is increased, the mass-transfer inside the channels is accelerated, generating a uniform spatial concentration distribution and localized supersaturated zones, that lead to the formation of smaller particles with narrower particle size distributions [52,85,86]. However, when the flow rate exceeds a certain limit, which can slightly differ from drug to drug, based on its physicochemical properties (e.g., lipophilicity and solubility in the S and AS mixture), micro-mixing becomes less homogeneous, accelerating the formation of larger particles characterized by broader size distributions [37,60,87]. It is clear from the results that the mixing stage is crucial in the determination of the final size of the particles, and that increasing the overall flow rate is beneficial only when complete mixing of the fluids is achieved.

**Figure 4:**
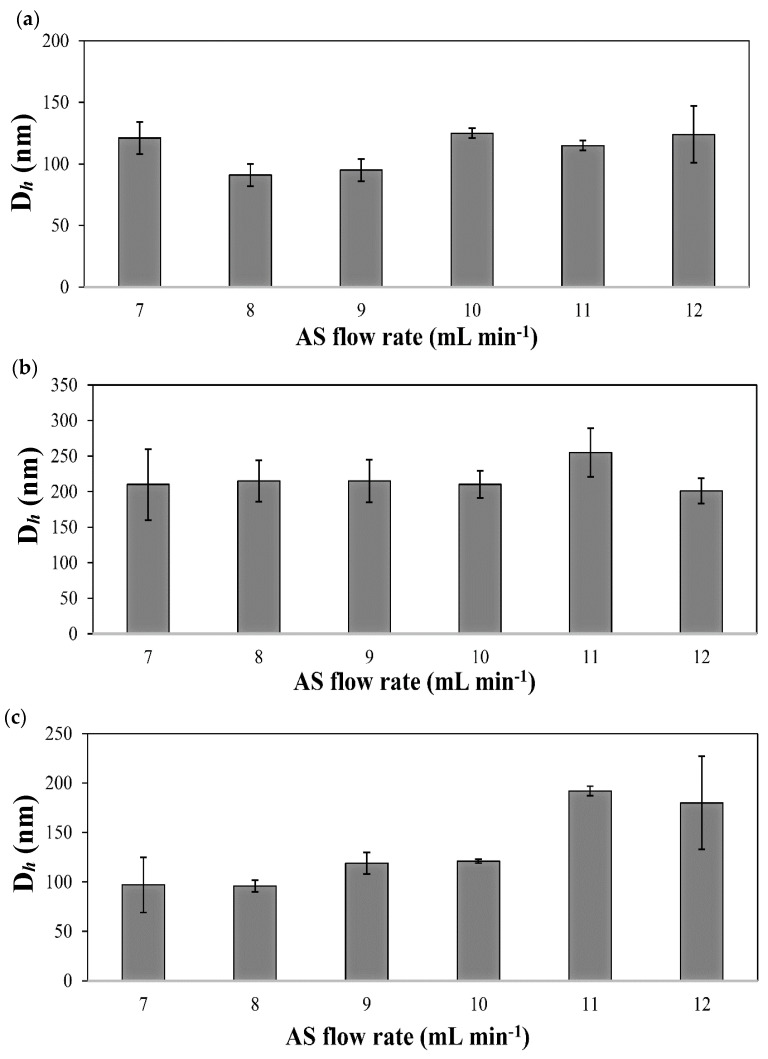
The effect of the variation of the AS flow rate on the hydrodynamic diameter (D*_h_*) of excipient-free pure (**a**) imatinib, (**b**) dasatinib and (**c**) tofacitinib nanoparticles produced by using a Y-shaped device, as measured by DLS at 25 °C.

Regarding the geometry of the channels (T- and Y-shaped), we anticipated differences in the flow pattern between the two shapes due to the difference in the inlet angle. In the T-shaped system, the inlet angle is 90° and the two fluids that meet in the junction are more likely to disrupt each other as they flow by creating stagnant zones. Contrarily, in the Y-shaped system, the inlet angle is 67.5° and hence at lower flow rates, the laminar flow of the S and AS is less disrupted [54]. Statistically significant differences in D*_h_* were observed between the two device geometries for IMA at the limits of low and high S/AS flow rate (0.05/0.5, 0.1/1.0, and 0.6/6.0 mL min^−1^/mL min^−1^) and for TOF at a flow rate of 0.5/5.0 mL min^−1^/mL min^−1^ (Figure 3a,c). In both cases, smaller particles were produced by the Y-shaped system. Furthermore, at a higher flow rate of 0.8/8.0 mL min^−1^/mL min^−1^, the D*_h_* difference between the two geometries was not observed for neither IMA nor TOF, probably due to enhanced mixing at a higher flow rates (higher Re value), reducing the effect of flow disturbance in the T-shaped device. In the case of pure DAS nanoparticles, the D*_h_* was not influenced significantly by the difference in the geometry of the devices under the same precipitation conditions (Figure 3b). Next, we kept the S flow rate constant at 0.5 mL min^−1^, varied the AS flow rate from 3.5 to 6 mL min^−1^ and studied the effect of the variation of the AS flow rate with respect to the S flow rate on the D*_h_* of the different PDNPs by using the Y-shaped device. The results are summarized in Table 2 and Figure 4.

Since the driving force behind the initiation of the precipitation process is the supersaturation of the drug solution in the S induced by the rapid mixing with the AS, we expected that higher AS flow rates will generate a homogenous nucleation that would lead to the formation of smaller particles [9,57]. However, this was not the case. Pure IMA and DAS nanoparticles did not show an apparent change in size with increasing AS flow rates. Pure TOF nanoparticles showed the opposite trend where faster AS flow rate resulted in a significant increase of the D*_h_* that could be attributed to incomplete mixing of the fluids with increasing water flow, resulting in the formation of larger particles with broader particle size distributions [61,88]. The latter PDNPs also showed larger PDI values compared to the IMA and DAS counterparts, possibly due to the more metastable nature of the nanonized TOF form which could be attributed to its lower lipophilicity [84].

Upon optimization of the process conditions, excipient-free pure kinase inhibitor nanoparticles were produced by using the T-shaped device by setting flow rates of 0.2 and 2.0 mL min^−1^ for the S and AS, respectively.

The Z-potential estimates the particle surface charge density, and it depends on the size of the particle and the concentration of charged moieties on the particle surface, which is also directly related to the pH of the medium. The absolute Z-potential value is associated to the physical stability of the colloidal system [89]. In this work, nanosuspensions were prepared in water with a pH value of ~5. Pure IMA, DAS and TOF showed negative Z-potential values of −18 ± 2, −18 ± 3 and −31 ± 3 mV, respectively. The negative Z-potential of all the PDNPs could be explained by the exposure of electronegative moieties (e.g., carbonyl) at the nanoparticle surface. In addition, the more negative value shown by TOF with respect to IMA and DAS would stem from the electronegativity of the nitrile functional group.

The physical stability of the free PDNPs was assessed by tracking the D*_h_* and the PDI over time by DLS; an increase of the D*_h_* indicates particle agglomeration and growth. Results are summarized in Table 3.

Results showed that the D*_h_* of pure IMA and DAS nanoparticles gradually grow after production. For example, after 7 days, the D*_h_* increased from 126 ± 5 and 209 ± 12 nm to 261 ± 4 and 540 ± 26 nm, respectively (Table 3). At the same time, only one size population was observed for both drugs throughout the whole experiment, which would be in line with the size growth of larger nanoparticles at the expense of smaller ones that underwent gradual dissolution, a phenomenon known as Ostwald ripening [75]. These results could be explained by the lower physical stability of excipient-free PDNPs than surfactant-stabilized ones and indicate that to prevent particle growth over time, products need to undergo drying immediately after production by means of a method that does not require the incorporation of additives (e.g., cryo/lyoprotectants in freeze-drying) and enables redispersion to regenerate particles of the original size, such as spray-drying [90]. In the case of pure TOF nanoparticles, the D*_h_* remained almost unchanged (Table 3). This finding could be attributed to the lower hydrophobicity and higher water-solubility of this compound compared to IMA and DAS [89].

**Figure 5:**
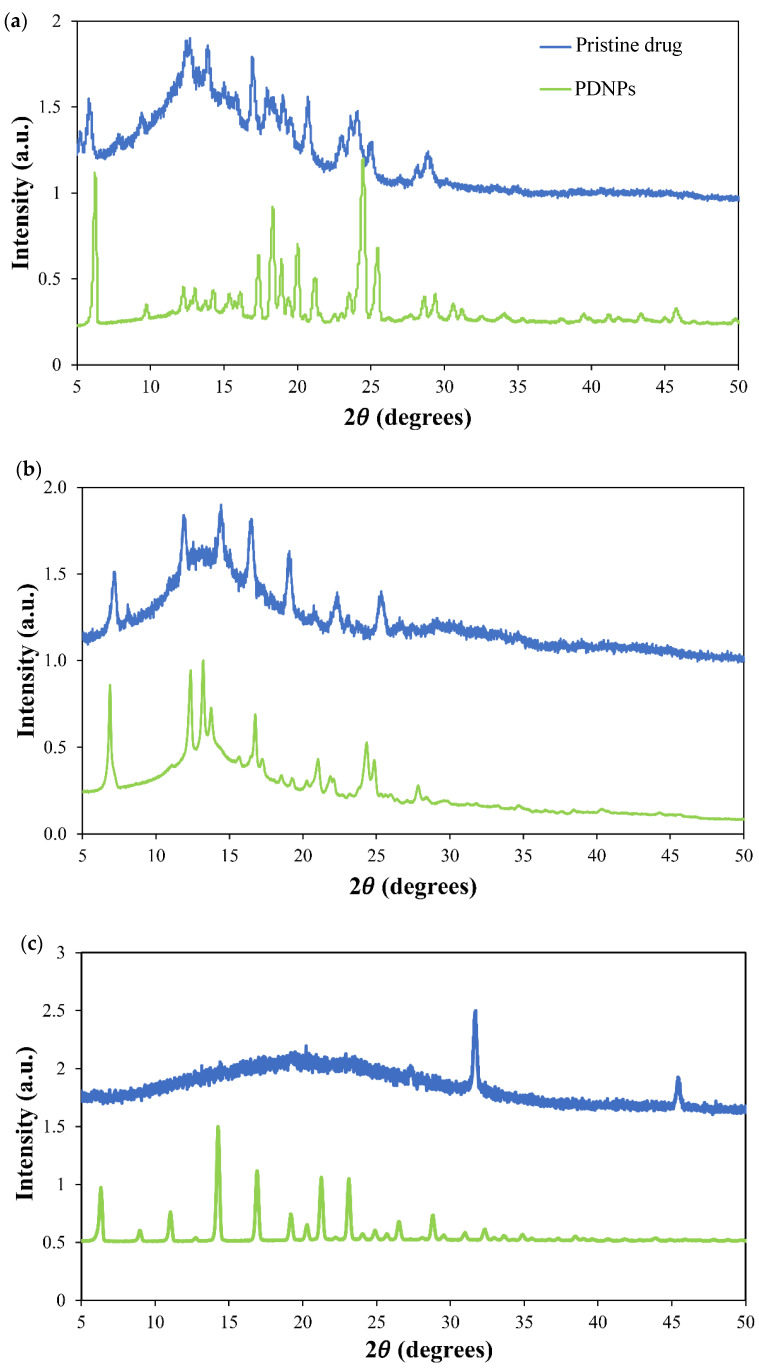
Powder X-ray diffraction patterns of (**a**) raw and nanonized imatinib, (**b**) raw and nanonized dasatinib and (**c**) raw and nanonized tofacitinib produced by using a T-shaped device at 25 °C.

The dissolution rate of PDNPs is not only governed by the size of the drug particle but also by its crystalline/amorphous state. Usually, the dissolution rate of amorphous drugs is faster than that of the crystalline counterpart [91,92]. In addition, drug amorphization can also result in greater saturation solubility. To assess the state of the three drugs upon nanonization, we analyzed their diffraction pattern by PXRD and compared it to the unprocessed counterparts. In general, the pristine drugs showed crystalline or semi-crystalline structure, and they underwent substantial amorphization upon nanonization, as clear from the broadening and, in some cases, the disappearance, of the diffraction peaks that are characteristic of the crystalline drug (Figure 5). For example, in the diffractogram of TOF, the raw drug showed a considerably high crystallinity as demonstrated by a series of sharp and intense diffraction peaks (Figure 5c) in accordance with literature [93]. Conversely, nanonized TOF formed a halo pattern typical of a substantially amorphous material, except for two peaks at 2θ = 31.7 and 2θ = 45.5 that were not observed in the XRD pattern of raw TOF (Figure 5c). The appearance of the new peaks (Figure 5c) suggests that this drug underwent changes during nanonization which may have led to the formation of a different polymorph [94].

It should be noted for PXRD and DSC (see below) analysis, PDNPs were freeze-dried immediately after synthesis, and both quenching and drying could explain the amorphous nature of the nanoparticles.

In the case of poorly water-soluble drugs, amorphous PDNPs could be preferred over crystalline ones to achieve faster dissolution rate under physiological conditions. On the other hand, amorphous drugs are usually less chemically stable [95,96].

**Figure 6:**
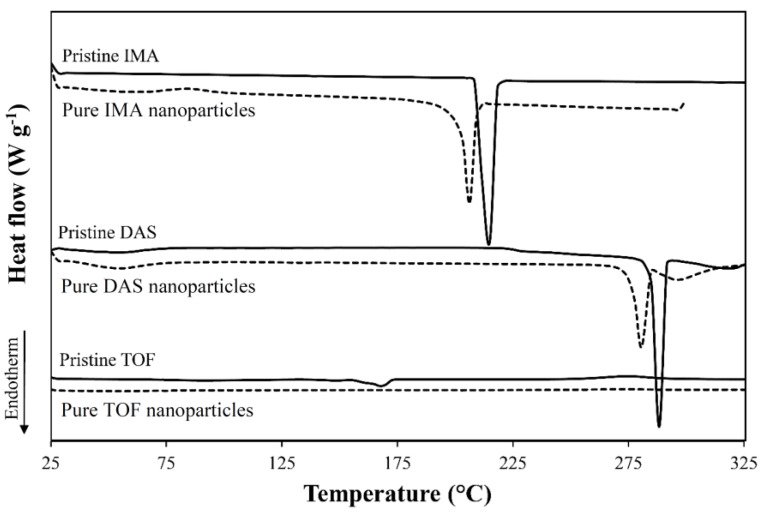
DSC thermograms of pristine and nanonized imatinib (IMA), dasatinib (DAS) and tofacitinib (TOF). Pure drug nanoparticles were produced by using a T-shaped device at 25 °C.

To support the PXRD results, the thermal behavior of the pristine and nanonized drugs was analyzed by DSC. Pristine IMA showed melting temperature (*T*_m_) at 215 °C (Figure 6). Pristine DAS and TOF showed two melting temperatures at 287 and 318 °C and at 148 and 168 °C, respectively, suggesting the presence of a mixture of different polymorphs [93,94,97]. The transitions in pristine TOF were very weak with very broad peaks (Figure 6). Nanonization led to a decrease of the *T*_m_ of IMA to 206 °C and to 280 and 295 °C for DAS. In addition, a decrease of the melting enthalpy (Δ*_h_*) of both drugs was observed (Table 4). These results confirm their semi-crystalline nature, though with a smaller degree of crystallinity than the pristine counterparts. Nanonized TOF did not show any thermal transition, which confirmed the very low crystallinity of these nanoparticles, in good agreement with PXRD data. The fact that IMA and DAS nanoparticles resulted in smaller amorphization extent compared to TOF counterparts could be attributed to the lower logP value of TOF. Zhu demonstrated that there is a good correlation between the drug logP value and the particle stability during nanoprecipitation, reporting that drugs characterized by lower logP values (<2) tend to have a more metastable nature in their nanonized form [84]. However, the exact mechanism responsible for the amorphization of crystalline/semi-crystalline drugs upon nanoprecipitation remains controversial [98].

**Figure 7:**
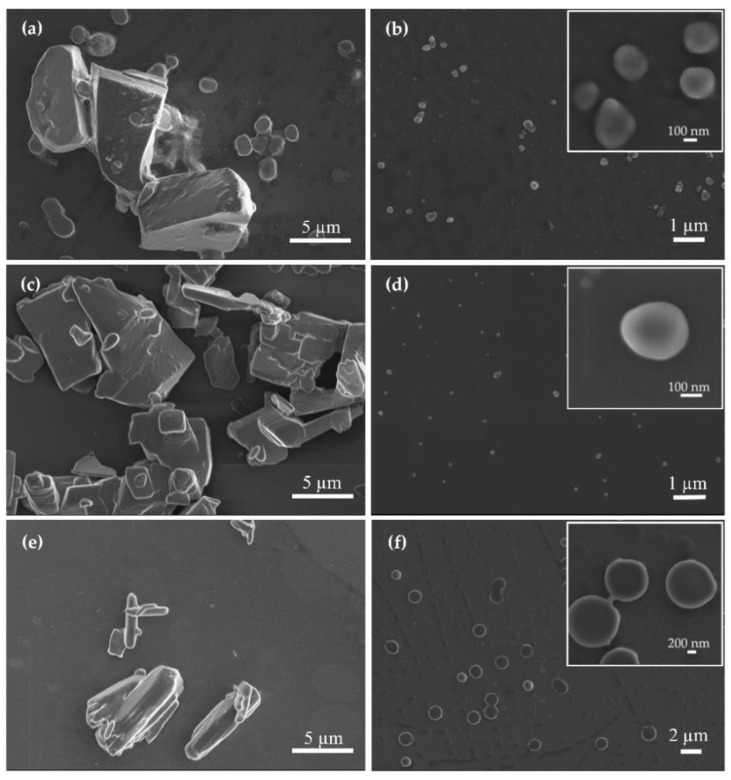
HR-SEM micrographs of (**a**) raw imatinib, (**b**) pure imatinib nanoparticles, (**c**) raw dasatinib, (**d**) pure dasatinib nanoparticles, (**e**) raw tofacitinib and (**f**) pure tofacitinib nanoparticles.

Then, the morphology of the nanoparticles was visualized by HR-SEM and compared to the unprocessed counterparts. Raw IMA (Figure 7a) and DAS (Figure 7c) showed irregular microparticles within a broad size range, suggesting some amorphousness. Raw TOF showed a more elongated morphology, characteristic of crystalline drugs (Figure 7e). All the PDNPs showed smooth, rounded morphology, consistent with their almost intermediate to high amorphous structure (Figure 7b,d,f) [99,100]. The size of the nanoparticles was significantly smaller and more uniform than that of the respective raw counterparts. The morphology of the particles suggests that the precipitation process inside our microfluidic device was fast, allowing the effective trapping of the drug nanoparticles in a less stable amorphous state [9,95,98,101]. These results further emphasize the advantage of the use of flow focusing techniques for the synthesis of additive-free nano-drugs of uniform size with enhanced saturation solubility and dissolution rates.

## 4. Conclusions

In this work, we produced surfactant-free PDNPs of three kinase inhibitors using a simple Si-based microfluidic device.

The first part of this work involved the study of the effect of the variation of different parameters in the microfluidic precipitation process on the D*_h_* of the synthesized nanoparticles. These parameters included the change in the geometry of the channels, the overall S/AS flow rate, and the ratio between S/AS flow rate with increasing AS flow rate. Results demonstrate that the nanoparticle D*_h_* was controlled and in the nanometric range with monomodal size distribution. In addition, changes in the S/AS flow rate have a strong influence on the D*_h_* and the size distribution of the produced particles. Additionally, differences in D*_h_* as a function of channel geometry are observed upon the synthesis of IMA and TOF nanoparticles at flow rates at which complete mixing between S and AS is not reached, which leads to the formation of particles with larger size in the T-shaped device. Following the optimization of the nanoprecipitation process, a comparative analysis of the results for each nano-drug and its pristine counterpart was performed. PXRD and DSC analysis revealed that drug nanonization led to a substantial decrease in the crystallinity with respect to the raw drug. In addition, HR-SEM confirmed that the particles are mainly amorphous, as demonstrated by their smooth and spherical shape and PXRD and DSC analysis. Overall, our results show the promise of this device setup to produce PDNPs of poorly water-soluble drugs. Ongoing research investigates the extension of this platform to the synthesis of different types of drug-loaded polymeric nanoparticles and hybrid ceramic/polymer nanomaterials, and the coupling of this production method with spray-drying to ensure the long-term physicochemical stability of the products.

## Figures and Tables

**Table 1:** The effect of the flow rate on the hydrodynamic diameter (D*_h_*) of excipient-free pure imatinib (IMA), dasatinib (DAS) and tofacitinib (TOF) nanoparticles produced by using T- and Y-shaped devices at a fixed drug solution concentration of 0.1% *w/v*, as measured by DLS.

Drug	S/AS Flow Rate, (mL min^−1^/mL min^−1^)	S/AS Volume Ratio	T-Shaped			Y-Shaped		
			D*_h_* (nm) ^1^ (±S.D.)	S.D. (nm) ^2^	PDI (nm) (±S.D.)	D*_h_* (nm) ^1^ (±S.D.)	S.D. (nm) ^2^	PDI (nm) (±S.D.)
IMA	0.05/0.5	1/10	196 (6)	75	0.13 (0.02)	143 (4)	53	0.18 (0.03)
	0.1/1.0		174 (9)	71	0.16 (0.01)	120 (12)	67	0.31 (0.01)
	0.2/2.0		133 (5)	60	0.30 (0.03)	146 (16)	50	0.24 (0.04)
	0.3/3.0		129 (5)	64	0.32 (0.02)	118 (7)	59	0.33 (0.05)
	0.4/4.0		91 (6)	37	0.30 (0.03)	107 (16)	60	0.30 (0.08)
	0.5/5.0		125 (4)	30	0.30 (0.03)	120 (3)	40	0.09 (0.02)
	0.6/6.0		160 (7)	66	0.20 (0.04)	90 (6)	38	0.30 (0.03)
	0.8/8.0		159 (12)	79	0.24 (0.02)	181 (7)	102	0.21 (0.00)
DAS	0.05/0.5		221 (10)	7	0.11 (0.01)	249 (14)	64	0.04 (0.01)
	0.1/1.0		223 (15)	49	0.20 (0.03)	162 (10)	60	0.15 (0.03)
	0.2/2.0		203 (6)	64	0.10 (0.00)	199 (6)	63	0.09 (0.02)
	0.3/3.0		184 (13)	44	0.02 (0.00)	181 (4)	52	0.07 (0.03)
	0.4/4.0		202 (39)	17	0.10 (0.05)	226 (26)	4	0.07 (0.02)
	0.5/5.0		213 (16)	5	0.05 (0.04)	210 (33)	9	0.04 (0.03)
	0.6/6.0		271 (21)	93	0.13 (0.00)	310 (10)	97	0.15 (0.07)
	0.8/8.0		243 (6)	72	0.12 (0.04)	316 (36)	82	0.05 (0.01)
TOF	0.05/0.5		188 (8)	54	0.31 (0.06)	174 (4)	78	0.20 (0.02)
	0.1/1.0		144 (10)	66	0.23 (0.05)	153 (6)	40	0.20 (0.02)
	0.2/2.0		99 (9)	22	0.43 (0.14)	108 (16)	26	0.34 (0.09)
	0.3/3.0		146 (15)	24	0.32 (0.07)	117 (16)	39	0.24 (0.07)
	0.4/4.0		130 (7)	52	0.32 (0.07)	114 (8)	65	0.30 (0.03)
	0.5/5.0		172 (17)	54	0.32 (0.00)	121 (10)	50	0.40 (0.08)
	0.6/6.0		148 (34)	28	0.50 (0.10)	143 (12)	70	0.32 (0.10)
	0.8/8.0		148 (20)	29	0.60 (0.20)	165 (17)	79	0.30 (0.06)

^1^ D*_h_* are the intensity distribution values expressed as the average of five runs (*n* = 5) ± S.D., as determined by DLS. ^2^ Standard deviation (S.D.) of each size population that is an expression of the peak width, as determined by DLS.

**Table 2:** The effect of anti-solvent injection rate on the hydrodynamic diameter (D*_h_*) of excipient-free pure imatinib (IMA), dasatinib (DAS) and tofacitinib (TOF) nanoparticles produced by using a Y-shaped device, as measured by DLS at 25 °C.

Drug	S/AS Volume Ratio	S/AS Flow Rate (mL min^−1^/mL min^−1^)	D*_h_* (nm) ^1^ (±S.D.)	S.D. (nm) ^2^	PDI (nm) (±S.D.)
IMA	1/7	0.5/3.5	121 (13)	48	0.21 (0.08)
	1/8	0.5/4.0	91 (9)	26	0.20 (0.06)
	1/9	0.5/4.5	95 (9)	31	0.20 (0.05)
	1/10	0.5/5.0	121 (2)	40	0.10 (0.02)
	1/11	0.5/5.5	115 (4)	40	0.30 (0.02)
	1/12	0.5/6.0	124 (23)	32	0.20 (0.06)
DAS	1/7	0.5/3.5	210 (50)	22	0.06 (0.04)
	1/8	0.5/4.0	215 (29)	7	0.04 (0.02)
	1/9	0.5/4.5	215 (30)	17	0.05 (0.01)
	1/10	0.5/5.0	210 (19)	33	0.09 (0.06)
	1/11	0.5/5.5	255 (34)	17	0.05 (0.03)
	1/12	0.5/6.0	201 (18)	7	0.04 (0.03)
TOF	1/7	0.5/3.5	114 (6)	52	0.40 (0.15)
	1/8	0.5/4.0	96 (6)	49	0.30 (0.06)
	1/9	0.5/4.5	113 (12)	65	0.40 (0.04)
	1/10	0.5/5.0	107 (26)	61	0.30 (0.03)
	1/11	0.5/5.5	209 (29)	105	0.40 (0.15)
	1/12	0.5/6.0	180 (47)	56	0.45 (0.10)

^1^ D*_h_* are the intensity distribution values expressed as the average of five runs (*n* = 5) ± S.D., as determined by DLS. ^2^ Standard deviation (S.D.) of each size population that is an expression of the peak width, as determined by DLS.

**Table 3:** Hydrodynamic diameter (D*_h_*) of excipient-free pure imatinib (IMA), dasatinib (DAS) and tofacitinib (TOF) nanoparticles produced by using a T-shaped device over time, as measured by DLS at 25 °C.

Drug	Time	D*_h_* (nm) ^1^ (±S.D.)	S.D. (nm) ^2^	PDI (nm) (±S.D.)
IMA	0 h	126 (5)	37	0.14 (0.05)
	2 h	234 (5)	67	0.10 (0.02)
	24 h	214 (15)	42	0.24 (0.05)
	2 days	216 (17)	43	0.30 (0.10)
	7 days	261 (4)	58	0.25 (0.02)
DAS	0 h	209 (12)	53	0.05 (0.01)
	2 h	300 (8)	74	0.04 (0.03)
	24 h	532 (39)	120	0.02 (0.03)
	2 days	500 (46)	110	0.20 (0.05)
	7 days	540 (26)	136	0.22 (0.01)
TOF	0 h	127 (5)	64	0.23 (0.01)
	2 h	101 (6)	33	0.20 (0.03)
	24 h	88 (2)	18	0.23 (0.03)
	2 days	111 (5)	40	0.20 (0.02)
	7 days	111 (6)	38	0.20 (0.02)

^1^ D*_h_* are the intensity distribution values expressed as the average of five runs (*n* = 5) ± S.D., as determined by DLS. ^2^ Standard deviation (S.D.) of each size population that is an expression of the peak width, as determined by DLS.

**Table 4:** DSC data of pristine and nanonized imatinib (IMA), dasatinib (DAS) and tofacitinib (TOF). Pure drug nanoparticles were produced by using a T-shaped device at 25 °C.

Drug	Form	T_m_ (°C) ^1^	ΔH_m_ (J g^−1^) ^1^
IMA	Raw	215	124
	Nanonized	206	106
DAS	Raw	287, 318	89/41
	Nanonized	280, 295	53/20
TOF	Raw	148, 168	3/52
	Nanonized	N.D.	N.D.

^1^ Determined in the heating ramp. N.D.: Not detected.

## Data Availability

The data presented in this study are available on request from the corresponding author.
